# Insights From a Child & Adolescent Mental Health In‐Patient Unit: Towards the Potential Development of Person‐Centred Practices

**DOI:** 10.1111/jcap.70067

**Published:** 2026-07-30

**Authors:** Christie Attard, Michelle Elliot, Paulann Grech

**Affiliations:** ^1^ University of Malta Msida Malta; ^2^ Queen Margaret University Edinburgh Scotland UK

## Abstract

**Problem:**

An increasing incidence in child and adolescent mental health difficulties calls for the development of effective person‐centred mental health services. This study aims to investigate the experiences of young persons, main caregivers, and healthcare professionals within a child and adolescent mental health in‐patient unit, with the aim to potentially develop person‐centred practices.

**Methods:**

A philosophical inquiry based on the SECI model within the concept of Ba was used. Three different methods were adopted, including the draw, write, and tell method with the young persons, semi‐structured interviews with the main caregivers, observations using the Workplace culture critical appraisal tool – revised, and the world café method with healthcare professionals.

**Findings:**

An indicative thematic analysis was completed collaboratively with two young persons, two main caregivers, and two healthcare professionals who previously took part in the data collection. Six themes emerged namely, power imbalance and communication, devaluing, boredom, support and feelings of isolation, getting out, and environment. Some themes were common for all three participant groups while others were specific to one group.

**Conclusions:**

It was evident that although some person‐centred moments were present, most notably, the formation of trusting relationships with some of the healthcare professionals, and feelings of support between healthcare professionals. However, several challenges toward the formation of person‐centred practice remain.

## 1 Introduction

There is an increasing incidence of mental health concerns for children and adolescents in European countries. The World Health Organisation (WHO) reported that 5.3% and 3.4% of adolescents between 15 and 19 years old experience anxiety and depression, respectively (WHO 2025). Suicide in young people also shows alarming rates with one of six of 15‐ and 29‐year‐olds die due to suicide or intentional self‐harm (Eurostat 2025). Furthermore, the WHO reported that adolescent mental well‐being has been consistently worsening since 2018 particularly in girls (WHO 2023). These statistics emphasise the need to work towards developing and creating effective person‐centred child and adolescent mental health services (CAMHS).

The aim of this study was to investigate the experiences of young people, main caregivers, and healthcare professionals working at a child and adolescent mental health in‐patient unit. This was done to explore the practices that were being followed within the unit. The following research questions will be explored in this research paper:

1. How are current care practices perceived by the young people receiving care, their main caregivers, and the healthcare professionals working within the unit?
2. What are the person‐centred moments experienced by young persons, their main caregivers, and the healthcare professionals?
3. How did these moments influence the experience of young persons, caregivers, and healthcare professionals?

A philosophical inquiry approach was used, which was based upon the SECI model (Nonaka 1994). This is a knowledge creation model that ‘emphasises the collaborative involvement of all the persons in the system’. To ensure the effectiveness of the SECI model, this process took place within ‘Ba’, which is a safe space where experiences and knowledge can be shared (Nonaka and Konno 2000). These principles are congruent with the principles of person‐centred research, where each person's values and beliefs are respected, and a sense of openness towards different ideas and possibilities is developed (Jacobs et al. 2017).

In this paper, the data generating and analysis methods are presented using the SECI model, and this is followed by a discussion of the themes and findings.

## 2 Methods

### 2.1 Study Setting and Participants

The recruitment for this study took place at a child and adolescent mental health in‐patient unit, between October 2023 and March 2024. Young persons between the ages of 12 and 18 years old can be admitted voluntarily or involuntarily if they are deemed to be at high risk of harm to themselves or others due to an acute mental health condition.

The sample comprised three groups: the young persons admitted to the in‐patient mental health unit, their main caregiver, and the multidisciplinary healthcare professionals. Following the principles of the SECI model, it was essential to gather tacit knowledge from everyone involved in the system and thus captured a comprehensive range of perspectives on the in‐patient experiences. A priori sample size of 15 young persons and main caregivers was determined prior to the initiation of the data collection. This decision was informed by reviewing average admissions records from the previous 2 calendar years. The number of healthcare professionals invited to participate in the study was 24, reflecting all professionals working on the unit on a full‐time basis at the time the study commenced.

This research was based upon the theoretical underpinnings of the SECI model; by following this process it was ensured that all persons within the service were included and encouraged to ‘share their personal knowledge’. Using such a process not only enabled the researchers to acquire as much knowledge as possible, but also fostered a sensitive approach towards all persons (Fayard 2003). This approach is congruent to the principles of person‐centred research, where research is done ‘with’ people rather than ‘for’ or ‘to’ them.

### 2.2 Socialisation – Data Collection

The use of the SECI model required participants to be actively involved in the process, thus methods used reflected the principles of participatory action research. These methods aimed to empower all persons to share their authentic experiences, whilst balancing power dynamics between the participants and the researcher (Baum et al. 2006). Different methods were used for each participant group to ensure that methods ‘were age appropriate and minimised participation burden’ (Table 1).

**TABLE 1:** Methods and participants.

Participant group	Methods used	Number of participants	Further information
Young persons	Draw, write, and tell	3 – Shared drawings and writings but declined interviews 1 – Participated by interview only 11 – Shared drawings and writings and participated in interview	Ages – 13–17 years Average days of admission – 14.2 days Voluntary admission – 12 Involuntary admission – 3
Main caregivers	Interviews	11 – Mothers of young person admitted 2 – Fathers of young person admitted 2 – Both parents of young person admitted participated	All legal guardians and biological parents
Health care professionals	Observations using the WCCAT‐R (Wilson et al. 2020) and World Café method	9 – Nurses including general and mental health nurses. 1 – Social worker 2 – Psychotherapists	Observations took place between October and December 2023. Professionals invited – 24 Accepted – 12

### 2.3 Externalisation – Data Analysis

Following the principles of the SECI model, the data analysis phase was completed collaboratively with two young persons, two main caregivers, and two healthcare professionals. Those involved were participants who shared their experiences in the first stage of the study. The data analysis process was facilitated with a focus group approach, during which an indicative thematic analysis was undertaken. The approach described as ‘Development’ (Jennings et al. 2018) was adopted. Initially, the aims, objectives, and research questions of the study were shared with all collaborators. A random sample number of transcripts was chosen, which were individually analysed and coded individually. These were then discussed and the main codes were generated and agreed by the whole group. The researcher then applied these codes to the rest of the transcripts, any additional codes or changes to the codes were then discussed and agreed to with all collaborators (Attard et al. 2025).

Throughout this process, it was vital to create Ba, to ensure that all collaborators felt valued and safe to share their thoughts about the generated data. The focus groups were held in a meeting place, which was neutral for everyone. In the first meeting, the researcher proposed a perspective of equality in the analysis group; although all participants came from different backgrounds and circumstances, during this group everyone held the same role as research collaborator. Furthermore, time was scheduled for the group to interact informally over coffee and food; enabling connection and conversation between group members in a more casual environment and without any predetermined titles. Although many collaborators held back in the first group, by the end of this phase of the project, the idea of a mutual relationship with mutual respect was reached. During the discussions all members were looking out for each other and each other's opinion, and none of the voices were diminished by more powerful voices. Some of the group members explained that they were happy that they were part of this collaboration as it gave them the opportunity to use a bitter life experience in a positive way. One of the young persons also commented that these discussions helped her to process a very difficult episode in her life, with people who could truly understand her experience.

### 2.4 Ethics

This study followed guidelines outlined in the Maltese Mental Health Act and was ethically approved by relevant ethics review boards.

## 3 Results

Data from all participants are presented below. For young persons, this includes both tablet‐based drawings and writings and interviews, and from the main caregivers and healthcare professionals, this data is represented from interview excerpts and writings of the focus group. Six themes were generated, some of the themes pertained to all three‐participant groups while others pertained to only one or two of the groups (Table 2).

**TABLE 2:** Themes.

Themes	Participant groups		
	Young persons	Main caregivers	Healthcare professionals
Power imbalance and communication	✔	✔	✔
Devaluing	✔	✔	✔
Boredom	✔	✔	
Support and feeling of isolation	✔	✔	✔
Getting out	✔		
Environment	✔	✔	✔

### 3.1 Power Imbalance and Communication

Issues related to power and communication were continuously referred to by both the young persons and their main caregivers in view of their relationship with the healthcare professionals. The theme of power imbalance and communication (or lack of) was also prevalent in the discussions between healthcare professionals' themselves.

**FIGURE 1:**
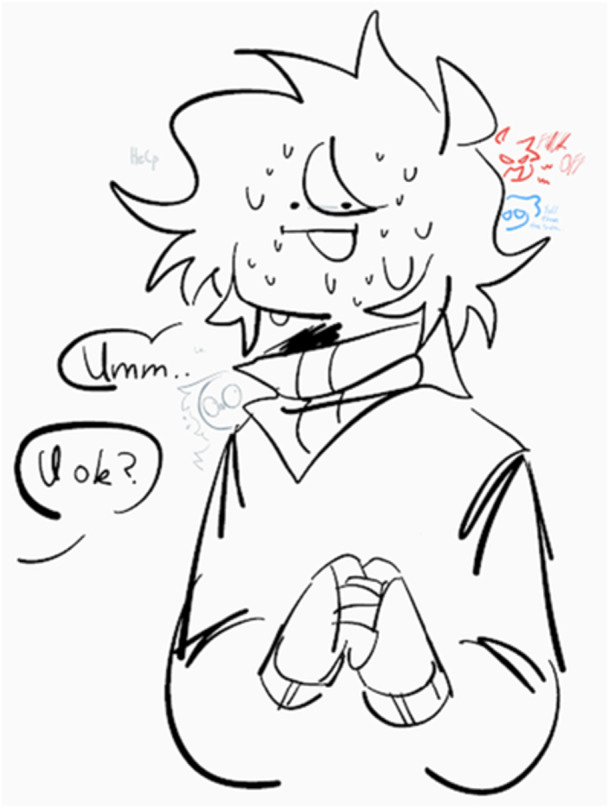
Emotional response to the admission process. [Color figure can be viewed at wileyonlinelibrary.com]

Young persons commented that ‘the only option (they) had whether I was being admitted voluntarily or involuntarily…It is like they are giving you a choice, but it is not really a choice’. Another young person presented the drawing below (Figure 1) as a picture of herself during the admission process. It portrays a sense of anxiety and fear, further explaining that ‘Red is me being mad and blue being upset. I was mad cause I was told nothing at all of why I was being sent there’.

Other young persons commented that they were given false information about the services offered at the Young People's Unit (YPU), such as the number of psychiatrist reviews or the number of therapy sessions. One of the young persons stated, ‘it filled me with a lot of false hope and didn't help my healing’. Main caregivers also showed concern about this, where the YPU was positively portrayed by the healthcare professionals., but they ultimately realised that not everything was necessarily true. Furthermore, for those experiencing a first admission, the fact that they were not reviewed immediately by their psychiatrist, felt like ‘nothing was happening’, which further exacerbated the feelings of helplessness.

Some of the main caregivers also experienced the lack of information during the admission process, with one of them stating that initially she was not sure to which hospital her child was going to be admitted. Conversely, the main caregivers of young persons who were benefiting from the community services, felt that they were involved in the decision of admission. Other main caregivers felt that although the decision of admission was not discussed with them, they trusted the healthcare professionals' decisions and agreed that it was the best way forward.

Some of the young persons also implied that the lack of communication between the professionals and themselves continued throughout their stay at the YPU. One youngster said:

…I'm so scared of asking anything or doing anything or saying something wrong the more I stay here the more I feel I'm gonna breakdown and cry, but I don't wanna cry I'm scared of crying I'm scared of showing any vulnerability.

Others commented on feeling vulnerable especially during ward rounds. These usually take place once per week, during which the young person will be reviewed by the consultant psychiatrist, the multi‐disciplinary team and their main caregivers. One young person described these ward rounds as ‘A lot of stress, cause a lot of people will be there. I was sweating’ with another adding ‘they ask you a lot of questions which usually you discuss one to one with a therapist’. While a main caregiver commented that she felt like she was being ‘assessed’. Some of the young persons felt that during the ward round they were not able to discuss their care plan, they were just asked questions and prescribed medications. Dependent upon the assigned psychiatrist, others commented that the interaction during the ward round was a positive and fruitful one, like ‘actual speaking human to human on the same level, not an interview’. Main caregivers also commented feeling intimidated during the ward round, with some finding it difficult to ask questions or clarify their concerns. Others commented that they used to be prepared and made sure that all their questions were addressed. As with the young people, it was noted that the experience of the interactions varied according to the consultant psychiatrist. Most caregivers commented that mostly they were not directly involved in the care plan, options available and decision‐making, but the plan and decision‐making process was clearly explained to them. One main caregiver commented ‘my heart sank. We were not prepared; we needed to consolidate and make sure that we are settled to welcome her back in the structure that we needed’.

Most caregivers expressed their concern about the lack of day‐to‐day feedback about the young person. Caregivers frequently shared that they were getting most the information from the young person him/herself, with one parent stating that ‘I had no other option. You have to literally accept it or you get angry and frustrated about it, which is not the time and place to do so’.

Healthcare professionals were described to be ‘caring’, ‘empathic’, and responsive when approached both by main caregivers and young persons, though there was a notable imbalance in communication. Main caregivers described the staff as ‘not forthcoming’, and thus they felt that they needed to initiate conversations around potential concerns or when requiring feedback. Young persons also shared mixed experiences: some commented that they could have ‘friendly conversations’ with nursing staff, others experienced staff to be ‘dismissive’ and ‘unapproachable’. Such inconsistencies suggest that access to effective communication was dependent upon each individual staff member, and on the initiative of the young person or caregiver, thus showing an uneven distribution of relational power.

Healthcare professionals noted that young persons communicate in distinctive ways like writing, drawing, and by presenting ‘difficult’ behaviour. Thus, skills like ‘being emotionally available’, being trustworthy and having the ability to create a safe environment were considered as crucial to enhance communication with the young persons. The ability to build a good therapeutic relationship was emphasised by healthcare professionals' multiple times. Some shared that this may be hindered by the ‘lack of teamwork with the multi‐disciplinary team’, and the use of formal language with the young persons. Furthermore, they suggested that nurses, in particular, can be more empathic towards the young person as they are the ones who spend the most time with the young persons. Conversely, communication was enhanced by sharing the professionals' experiences with the young person when and if appropriate, the use of the keyworker system, and by being ‘emotionally available’.

When discussing communication between the different participant healthcare professionals, it was evident that several barriers were present. One of the main barriers was that the healthcare system follows the medical model, thus the perception of the consultant psychiatrist having the most power in the professional hierarchy remained. This was previously noted during the ethnographic observations undertaken by the researcher. Moreover, nurses (who formed the majority of participants) felt they had no say in the development and the changes of the young person's care plan, even if they disagreed. They further commented that feedback from nurses was not taken seriously, and this usually resulted in lack of motivation and feelings of not being trusted professionally. Since young people were not regularly involved in the care plan, healthcare professionals felt a need to act as their advocates. Furthermore, they stated that most often there was a lack of appropriate handover between different professionals, and thus not all professionals had the full picture of the progress in the care plan until the day of the ward rounds. As noted by young persons and caregivers, healthcare professionals explained that when they previously worked with young persons in the community they were more involved in the formulation and the follow‐up of the care plan, mainly because they were assigned keyworkers to the specific cases, evidently this enhanced the experience of both the young persons and caregivers.

On a ward level, nurses felt that power was shared between nurses on the ward, conversely, regarding issues that involved higher management they felt that they were ‘treated as numbers’. Healthcare professionals stated that mostly they were not involved in the policy‐making or any changes in existing policies, once again portraying that although they were directly affected, they were not consulted as most decision‐making on a higher level was done solely by the hospital's higher management.

### 3.2 Devaluing

The data from the experiences of the young persons admitted at the YPU reveals issues of systemic rigidity (i.e., a sense of rigidity within the system), that lead to feelings of emotional neglect, disempowerment, and devaluing. One young person commented that they were ‘scared of crying, I'm scared of showing any vulnerability’, suggesting that the young person was not feeling that the unit offered a safe space to express their emotional distress and vulnerability. Another young person reflected that they felt that there was an expectation to get better which made the admission at the YPU ‘hard’. Some young persons stated that they felt that they were ‘dismissed’ and judged by healthcare professionals. One young person stated that:

There was a lack of care for the patients, it felt like we were more entertainment than patients sometimes.

**FIGURE 2:**
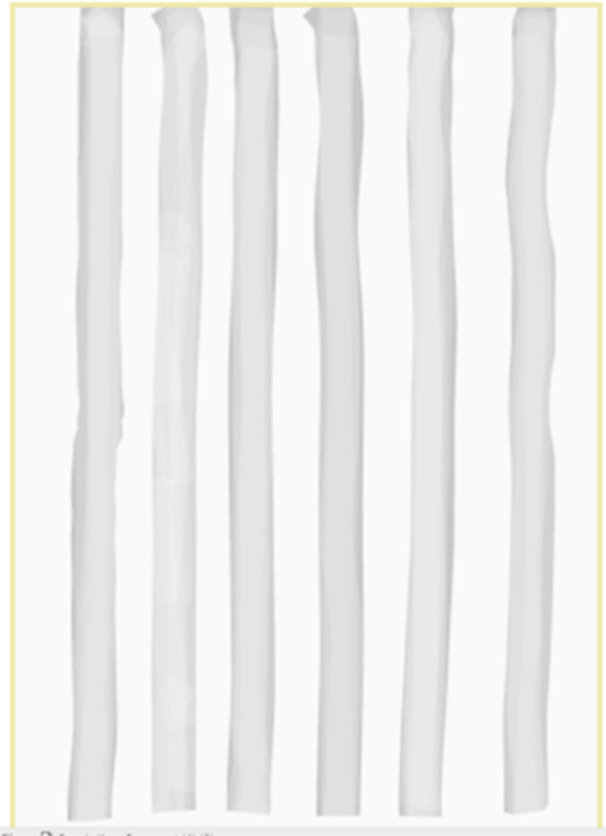
Just like a prison. [Color figure can be viewed at wileyonlinelibrary.com]

They clarified this comment by stating that there was constant lack of communication and interaction throughout the day. Other young persons compared YPU to a ‘prison’ (Figure 2) or ‘a very bad hotel’, due to the very strict schedules and lack of autonomy (timing of showers), and feeling that they were constantly being watched. One young person commented that they felt that they ‘were being punished for not doing well’. Experiences of misgendering and lack of gender‐affirming care were pointed out with one young person saying ‘they consistently used the wrong pronouns even when I corrected them’. Such concerns highlight how institutional routines led to feelings of diminished control and agency where young people could not maintain autonomy over the decision‐making for basic aspects of daily living.

Main caregivers described feeling emotionally overwhelmed and unsupported, with one describing the admission as ‘a big trauma’, with no opportunity to debrief or to ask for support. These feelings were consistent in the period when the young persons were discharged from the unit, with one caregiver stating ‘when they (young person) went back home it felt like they were forgotten’, as no further support was offered. Furthermore, the consistent lack of communication left parents out of important decisions related to their children. One caregiver stated that they were not informed about the involvement of child protection services, which left them with feelings of betrayal and distrust in healthcare professionals, moreover, feeling that their role as caregivers was diminished. Other caregivers felt that they were being judged as ‘everyone was blaming us’ and that they ‘did not matter as parents’. Such feelings were also highlighted by healthcare professionals, who at times felt that decisions were made irrespective of the feedback given, which resulted in feelings of not being trusted.

### 3.3 Boredom

Most young persons perceived time as dragging, with multiple respondents claiming that they felt that time slowed down due to the absence of engaging activities: ‘seconds feel like minutes’. Caregivers pointed out that given that this is a mental health in‐patient unit, this does not make sense as having a lot of idle time usually results in worsening of symptoms. The lack of structure was also described as passive and unproductive, creating a sense of stagnation; both young persons and main caregivers implied that the days were repetitive with no therapeutic value.

**FIGURE 3:**
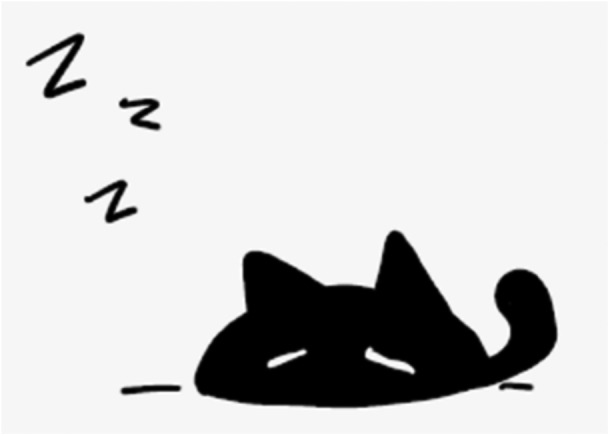
When boredom hits.

Statements made by caregivers expressed concern over this perceived therapeutic void, with one participant claiming: ‘You notice that from living her life normally, she is now sitting on a sofa facing a door for the entire day’. Caregivers also pointed out that young persons need stimulation, something which was lacking at the YPU. Other caregivers and young persons commented on the tendency to sleep to pass the time (Figure 3), with some young people complaining that even though the health care worker used to wake them up from napping, they still used to fail to engage them in activities.

Some young people and caregivers offered reflections on positive moments and opportunities, such as the time spent in the unit's classroom, a space where they could engage in educational and creative activities. Other young persons commented that the things done in class were too basic and thus they did not manage to learn anything. There were also independent staff who took the initiative to organise group activities, but these were not consistent and not part of a daily structure. Furthermore, some caregivers and young persons viewed the lack of activities as a positive thing, with one caregiver claiming that this gave the opportunity to her child to focus on studying for her upcoming exams. Others commented that they could spend time doing things that they enjoy like reading and self‐reflection.

### 3.4 Support and Feelings of Isolation

**FIGURE 4:**
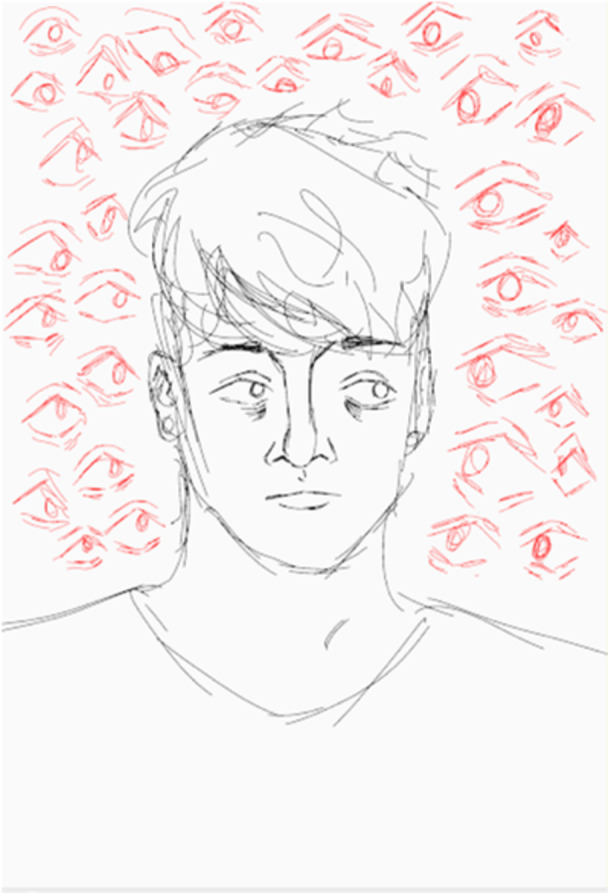
Watched. [Color figure can be viewed at wileyonlinelibrary.com]

Young people's experiences of the admission at the YPU highlighted a sense of isolation and relational disconnection. Some describe feelings of fear, emotional overwhelm, and pressure to recover, with one young person stating: ‘Being here is hard. It feels as though I'm under the constant pressure to just feel…better’. This may have resulted in feelings of guilt and frustration to an already difficult experience. Most young persons commented negatively about the elevated supervision, with some referring to it as ‘being followed by a security’ and being watched all the time (Figure 4) without offering any kind of support. Although they understand the intended purpose of elevated supervision, most still describe it as being intrusive, ineffective, or dehumanising. One participant compared it to being treated ‘like an animal in a zoo’, which shows feelings of loss of autonomy, dignity, and privacy, while still feeling a sense of loneliness and helplessness.

Feelings of isolation were commonly portrayed in the data, mainly in response to the restrictions on contact with friends, extended family, and restrictions over the use of technological devices. Young persons commented that only parents were allowed to visit and a typical visit lasted for a very short time during the day. Telephone contact may have followed at different times of the day. Such restrictions impacted the potential for support that young persons could receive from friends and extended family members. The intention of such policies was to manage risks associated with safety and confidentiality during hospital visits and use or access to technological devices.

Emotional support by healthcare professionals was mostly individual‐dependent rather than system‐driven. Some young persons stated that some nursing staff took time to listen and were able to build a connection with them. In the words of one young person, ‘some of the nurses…gave me a sense of humanity’. Conversely, others had negative experiences and perceptions of detachment and inattentiveness from healthcare professionals. Most young persons experienced an absence in therapeutic engagement in general, feeling that the system was rigid and did not always cater to their individual needs.

At times, these feelings of isolation and lack of support were improved with peer relationships formed within the unit. Young persons leaned on one another for emotional validation, this portrays the importance of shared lived experiences in the therapeutic process. Furthermore, some young persons commented that nurses ‘used to talk to us like they were our friends’ thus being able to build positive relationships with them.

Data from main caregivers showed two opposing perspectives, one where caregivers expressed feelings of gratitude and trust toward professionals, and another where they shared experiences of emotional isolation and gaps in communication and support. Once again, the level of interaction between the healthcare professionals and the caregiver was highly dependent on the individual professional. Some caregivers valued genuine, personal connections with staff members with one commenting ‘There were two nurses, I consider them as friends’. Experiencing therapeutic interactions that conveyed empathy and kindness had a significant positive impact, as these caregivers felt that they were acknowledged, valued, and re‐assured. Furthermore, some caregivers reported that they always felt that they were welcome and were encouraged to communicate openly with the healthcare professionals.

Conversely, other caregivers presented stories about their sense of exclusion and emotional detachment as they did not find it easy to approach the professionals and discuss any concerns. A lack of formal support and education to the caregivers was identified. One caregiver commented that during the admission she was so overwhelmed by other responsibilities that getting support at that time did not feel essential. This lack of support was described by some caregivers as emotionally taxing and ‘isolating’. Although all caregivers agreed that an admission to the YPU was the best decision at the time, the emotional weight of such decisions was still felt.

Feelings of lack of support were also identified by the healthcare professionals. They perceived the management as reactive rather than supportive, feeling at risk of being blamed and scrutinised if an incident occurred. The lack of support was also perceived in the general absence of adequate training, particularly in critical areas like risk assessment. Such lack of training often resulted in anxiety and reduced confidence. Additionally noted were frustrations with the barriers to liaising with other agencies, which contributed to delayed discharges. Most healthcare professionals claimed there are no ‘clear indicators of expectations’ thus they were held to vague or unrealistic standards alongside limited or inconsistent support structures.

### 3.5 Getting Out

Some young persons perceived their time at the YPU as time wasted and their stay was ineffective. One young person commented that the time at YPU was ‘a waste of time’, marked by boredom and disruption of usual activities like going to school. Strikingly, several young persons admitted that they lied about their mental health and emotional wellbeing in order to be discharged. ‘I felt like I got better at lying’. This may suggest that young person's felt that they were unable to express their emotions and feelings due to a fear of judgement and prolonged admission. These reflections may also reflect a limited understanding of the reason for admission to the YPU, perhaps contributing to the determination that there was no need to be on the unit. Another young person indicated that they were deeply affected by the admission experience: ‘they did one good job because I do not want to get back in there’.

### 3.6 Environment

Although most comments from young persons and their main caregivers about the physical environment were negative, some positive aspects were highlighted, including: pleasant food, rooms that had a lot of light and were described as ‘warm and cosy’. Some young persons commented that the seasonal decorations made the place look better. Others showed general tolerance towards the environment, stating that although it was not the ‘worst’ environment, they would not want to be there again. Some main caregivers ascribed the characteristics of the physical environment to their expectations of a mental health in‐patient unit or sterile hospital: lack of colours and lack of homeliness.

Young persons explained that the ‘the things (in the unit) are pretty old, I do not think that the manage the area really well’. One of the caregivers also remarked that ‘the environment is not warm and homely…it is very cold and clinical’. Another youngster commented that there is ‘way too much sound going on all the time’, as the unit is relatively small there are limited spaces where quiet time alone might be possible.

Some of the main caregivers shared that they were not shown around the entire unit, thus made them feel a sense of discomfort and anxiety as they were not able to physically see where the young persons were sleeping and spending their time. Healthcare professionals also identified similar environmental issues including: the lack of space, the lack of warm welcome, and lack of appropriate furniture. They pointed out that several improvements could be made, like increasing the resources and material for therapeutic activities, developing areas where they could hold activities, the development of a waiting room for parents and improvement in the CCTV system. Such changes were discussed or requested mostly by nursing staff but never came to an avail. Some positives were also pointed out, including the presence of safety pods, bean bags, and ‘the calm down box’ which all helped to create a more calming and comfortable environment.

## 4 Discussion

Issues of power and communication align with recent literature related to child and adolescent mental health services. Feelings of not being involved in the care or being misinformed may exacerbate feelings of anxiety and helplessness as seen in the accounts given by young persons and main caregivers. Spencer et al. (2019) noted that clinical engagement and methods of communication can highly contribute to episodes of acute distress. The experiences highlighted several deficits in communication, like lack of communication and information about the admission, misinformation about the services that were going to be offered that lead to ‘false hopes’, delays and lack of information about the psychiatric reviews. Furthermore, this appeared to make young persons feel that they were unable to make informed decisions about their care and had little to no control over their care and progress. Such experiences can be detrimental when receiving care as they often lead to lack of trust in the system leading to poor engagement and poor therapeutic alliance (Roach et al. 2025; Ahuja and Williams 2010; Moran and Gutman 2021). Feelings of coercion in child and adolescent mental health care, for children who are voluntarily or involuntarily admitted, are similar to those seen for adults (Nyttingnes et al. 2018). Healthcare professionals commented about the medical model hierarchy, where most clinical decisions lie within the responsibilities of the consultant psychiatrist. This indicates a sense of power imbalance within the team. The feelings of being unheard and not professionally valued can results in lack of interest in the job and most often ultimately burn out (Fond et al. 2022).

All participant groups in their own ways expressed feelings of disempowerment and devaluation, which have impacted the therapeutic environment. A young person's fear of showing their emotions and vulnerability implies that the ‘essential ingredients of a therapeutic milieu’ (Clark and MacLennan 2023, 7) have not been enacted on the YPU. Caregivers and young persons highlighted the lack of consistent meaningful structure and therapeutic activities, which resulted into feelings of boredom. Such findings were consistent across other young person's in‐patient services (NSPCC 2024). The feelings of boredom may not only be attributed to the lack of activities, as studies show that feelings of boredom are common during the adolescent years and can be ‘functional for psychological growth’ (Biolcati et al. 2018, 304). The constant positive comments about the time spent in the classroom underscore the value of having structured activities on the ward.

Feelings of not being supported and feeling overwhelmed were also reported by caregivers. Offering emotional support to caregivers is essential, as it may directly impact their ability to support the young person. Furthermore, caregivers at times were not seen as partners in care thus at times were not directly involved in the planning. Lack of feedback mostly resulted in feeling of anxiety and isolation in caregivers, with some commenting that they felt that they could not raise their concerns as they feared possible negative impact on the young person. Issues of lack of information and communication were consistent with other studies (Tas et al. 2010), furthermore such feelings may have exacerbated feelings of lack of support (Merayo‐Sereno et al. 2021).

While some elements of person‐centred practice exist within these findings, including the trusting relationships formed between the young people, parents, and staff, empathic encounters, and the sense of support between groups of staff working on the ward, there are systemic issues that are prohibiting such practices from functioning. Looking at the Person‐centred Practice framework developed by McCormack and McCance (2017) it was noted several lacunas in the different stages presented in this framework. Although some of the prerequisites may be present, with trained staff, for example, the clear feelings of not being professionally valued and heard, may inhibit the successful development of person‐centred practices. This is also portrayed in the care environment where once again the rigid schedules, monotonous environment, and evident use of the medical model create an environment which limits empowerment and opportunities. Furthermore, while some individual healthcare professionals seem to adopt certain person‐centred processes like maintaining effective communication, sharing decision‐making, and being sympathetically present, such processes are not systemically embedded within the system. When looking into this, it is evident that young persons, parents, and health care professionals within the in‐patient unit do experience sporadic person‐centred moments, such as the trusting relationships that some healthcare professionals develop with young persons and parents, the support shared between nurses, and positive experiences during the ward rounds. Though several challenges to the development of person‐centred practice were noted, including the difficulties in the physical environment, lack of resources and the lack of a sense of teamwork within the team (Kayes and Papadimitriou 2023).

Throughout this study, the researcher worked intentionally to maintain ‘Ba’. It was incorporated in different ways in the different stages: by the use of the tablets, by the spaces chosen for the interview, and the environment created during the world café method. One can note that generally no technology devices were allowed on the ward, but this study offered the opportunity to use such devices as a communication tool. Furthermore, paying attention to the physical environment further emphasised the positive impact of this, as highlighted in the person‐centred practice framework (McCormack and McCance 2017). Ba was created by allowing participants to openly share their ‘feelings, emotions, and experience’ (Nonaka and Konno 2000). Highlighting this opportunity is essential as at times young person's felt that this sense of safety was not experienced during their time at the YPU. In the data analysis stage, Ba was created by encouraging reflection and discussion of the group collectively. During this stage it was clear the connections between all persons within the group were developed, ‘creating a sense of safety and understanding’.

## 5 Implications on Practice

These findings highlight the need to strengthen person‐centred practices within the child and adolescent mental health in‐patient unit. Maintaining constant communication between all persons involved within the service is vital. Ensuring effective communication between the caregiver, young persons, and healthcare professionals ensures parent satisfaction, as well as improved professional wellbeing (Marino et al. 2023). Furthermore, proactive and transparent communication can reduce feeling of helplessness and coercion.

Continuous support and training to healthcare professionals is key in the development of person‐centred practices. Moreover, enabling an improvement in the physical environment of the unit, as well as the resources provided may help in developing more collaborative structures and minimise the existing rigid routines. The development of a holistic programme with structured recovery‐focused activities will help in promoting agency and increase therapeutic engagement. Lastly it is essential to actively involve the caregivers, not just by including them in the care planning and decision‐making but also by supporting them and psycho‐educate them about ways that they can support the young persons in care.

## Conflicts of Interest

The authors declare no conflicts of interest.

## Policy on Using ChatGPT and Similar AI Tools

No AI tools were used in the writing, development, and editing of this manuscript.

## Data Availability

The data that support the findings of this study are available from the corresponding author upon reasonable request.
